# Large Language Model–Assisted Discharge Instructions for Diabetic Kidney Disease: Comparative Evaluation Study

**DOI:** 10.2196/100792

**Published:** 2026-11-13

**Authors:** Miaomiao Cheng, Huiru Wang, Qi Zhang, Jun Qin, Lei Gong, Ling Guo, Xiaoyan Xiao

**Affiliations:** 1 Department of Nephrology, Qilu Hospital of Shandong University, No.107, Wenhua West Road, Lixia District, Jinan, Shandong, China, 86 18560083877; 2 Healthcare Big Data Research Institute, Cheeloo College of Medicine, Shandong University, Jinan, Shandong, China; 3 Department of Endocrinology, Qilu Hospital of Shandong University, Jinan, Shandong, China

**Keywords:** artificial intelligence, diabetic kidney disease, discharge instructions, large language model, patient education

## Abstract

**Background:**

Discharge instructions are an important component of the transition from hospital to home for patients with diabetic kidney disease (DKD), whose care often involves multiple comorbidities, medications, and stage-specific management needs. However, producing complete, accurate, clear, and personalized instructions can be challenging for junior physicians.

**Objective:**

This study aimed to evaluate whether large language model (LLM) assistance improves the quality of DKD discharge instructions produced by junior physicians and whether its benefits vary according to chronic kidney disease (CKD) stage.

**Methods:**

The study included 101 hospitalized patients with DKD. Discharge instructions for each patient were initially drafted by a group of junior residents (resident group); subsequently, another group of residents at the same level, with the assistance of DeepSeek-V3.2 (AI-assisted group), generated a separate set of discharge instructions for the same 101 patients, resulting in a total of 202 documents. Four clinical experts, including 2 nephrologists and 2 endocrinologists, independently scored both versions of the discharge instructions using a 5-point Likert scale across 5 dimensions: completeness, accuracy, safety, clarity, and personalization. Interrater reliability was assessed using the intraclass correlation coefficient (ICC).

**Results:**

Interrater reliability was excellent in both the resident group (ICC=0.817, 95% CI 0.763‐0.864; *P*<.001) and the AI-assisted group (ICC=0.860, 95% CI 0.816‐0.897; *P*<.001). Compared with resident-only instructions, AI-assisted instructions received significantly higher scores across 5 evaluation dimensions and the overall evaluation (all Bonferroni-adjusted *P*<.001), with large effect sizes (*r*=0.741‐0.927). In the resident-only group, CKD stage was negatively correlated with accuracy, safety, clarity, and overall quality (Spearman ρ=−0.33 to −0.37; all *P*<.001), whereas no significant correlations were observed for most dimensions in the AI-assisted group (|ρ|<0.15; *P*>.05). The proportion of cases achieving a ≥1-point improvement in overall quality was 99% (100/101; 95% CI 94.6%‐100.0%).

**Conclusions:**

LLM assistance improved the completeness, clarity, and personalization of DKD discharge instructions generated by junior physicians, with advantages greatest in complex cases. A human-AI collaborative model is a promising approach to reduce documentation burden.

## Introduction

Diabetic kidney disease (DKD) is a common and clinically important complication of diabetes and remains a major cause of chronic kidney disease (CKD) and kidney failure worldwide [1,2]. DKD is frequently accompanied by multiple comorbidities and complications, including hypertension, anemia, cardiovascular disease, electrolyte disturbances, and metabolic abnormalities, resulting in substantial heterogeneity in treatment and self-management needs [3]. Contemporary CKD guidelines emphasize individualized risk assessment, medication stewardship, management of complications, and patient-centered care across different stages of CKD [4,5]. The complexity of these requirements becomes particularly important at hospital discharge, when patients and caregivers must transition from inpatient treatment to long-term self-management.

The transition from hospital to home is a critical point in chronic disease management. For patients with diabetes and CKD, an appropriate discharge plan should address medication reconciliation, glucose monitoring, recognition and management of hypoglycemia, dietary and lifestyle recommendations, follow-up arrangements, and disease-specific warning signs [5]. Current diabetes care standards recommend that care be individualized and that patients receive education and support relevant to their treatment plans [5]. However, translating complex clinical information into discharge instructions that are simultaneously accurate, comprehensive, understandable, and tailored to an individual patient remains challenging. Junior physicians, in particular, must integrate clinical diagnoses, laboratory abnormalities, medication regimens, comorbidities, and follow-up requirements while working under substantial documentation demands. Deficiencies in information organization or clinical experience may result in omissions, excessive technical language, or insufficient personalization [6-9].

Large language models (LLMs) have recently emerged as potential tools for clinical documentation and patient communication. These models can summarize clinical information, reorganize medical content, and generate patient-oriented text, and recent studies have demonstrated their potential for producing or transforming discharge-related materials [10-15]. For example, generative AI has been evaluated for transforming inpatient discharge summaries into more patient-friendly formats [13], while other studies have examined the quality and safety of LLM-generated patient-centered discharge instructions [14]. More broadly, systematic reviews published in 2025 suggest that LLMs are increasingly being investigated in real-world clinical workflows, including documentation and communication, although evidence remains limited regarding implementation, generalizability, and appropriate human oversight [9,10]. Importantly, recent evidence also highlights that the performance of LLMs can vary according to task complexity and clinical context, supporting the need to evaluate human-AI collaboration rather than treating LLMs as autonomous substitutes for clinicians [9,10].

Despite these advances, evidence regarding LLM-assisted discharge instruction generation for patients with complex chronic diseases remains limited. Previous studies have largely focused on either fully automated LLM-generated materials or general clinical documentation, whereas the potential value of an assistive workflow in which junior physicians use an LLM to generate a draft and subsequently review and finalize the content has been less systematically evaluated. This question is particularly relevant to DKD because increasing CKD stage is often accompanied by greater medication complexity, more frequent complications, and more individualized management requirements. Whether LLM assistance can maintain the quality of discharge instructions as clinical complexity increases therefore remains unclear.

Therefore, this study aimed to compare the quality of DKD discharge instructions produced by junior physicians alone with those produced by junior physicians assisted by an LLM, using the same patient cases for direct comparison. We evaluated 6 dimensions of document quality—completeness, accuracy, safety, clarity, personalization, and overall quality—and further examined whether the relative performance of the 2 approaches varied across CKD stages. We hypothesized that LLM assistance would improve the overall quality of discharge instructions while providing particular benefits in more clinically complex cases.

## Methods

### Study Design

This study used a parallel-group comparative design to evaluate differences in quality between 2 methods of generating discharge instructions (resident group vs AI-assisted group). A total of 101 hospitalized patients with DKD were enrolled. The discharge instructions for the resident group were derived from actual documents that had been written by a group of junior residents during their routine clinical practice and retrospectively collected. In the AI-assisted group, another cohort of junior residents with comparable clinical training and experience drafted discharge instructions for the same 101 cases with the assistance of DeepSeek-V3.2, resulting in a patient-matched design (a total of 202 discharge instructions). There was no overlap between the 2 groups of residents, and neither group had access to the other’s output. This parallel design was adopted to minimize potential bias from memory traces or learning effects that might arise if the same group of physicians participated in both groups, thereby ensuring the comparability of the 2 document-generation strategies.

In the AI-assisted group, residents provided the AI system with anonymized patient data (including basic test results and treatment plans) and instructed DeepSeek-V3.2 to generate an initial draft. Subsequently, residents reviewed and revised the draft based on their clinical judgment before finalizing it. The evaluation experts were not blinded to the group assignments, as the AI-assisted discharge instructions were easily identifiable due to their highly structured, patient-friendly format. The whole flow chart is shown in (Figure 1).

**Figure 1.**
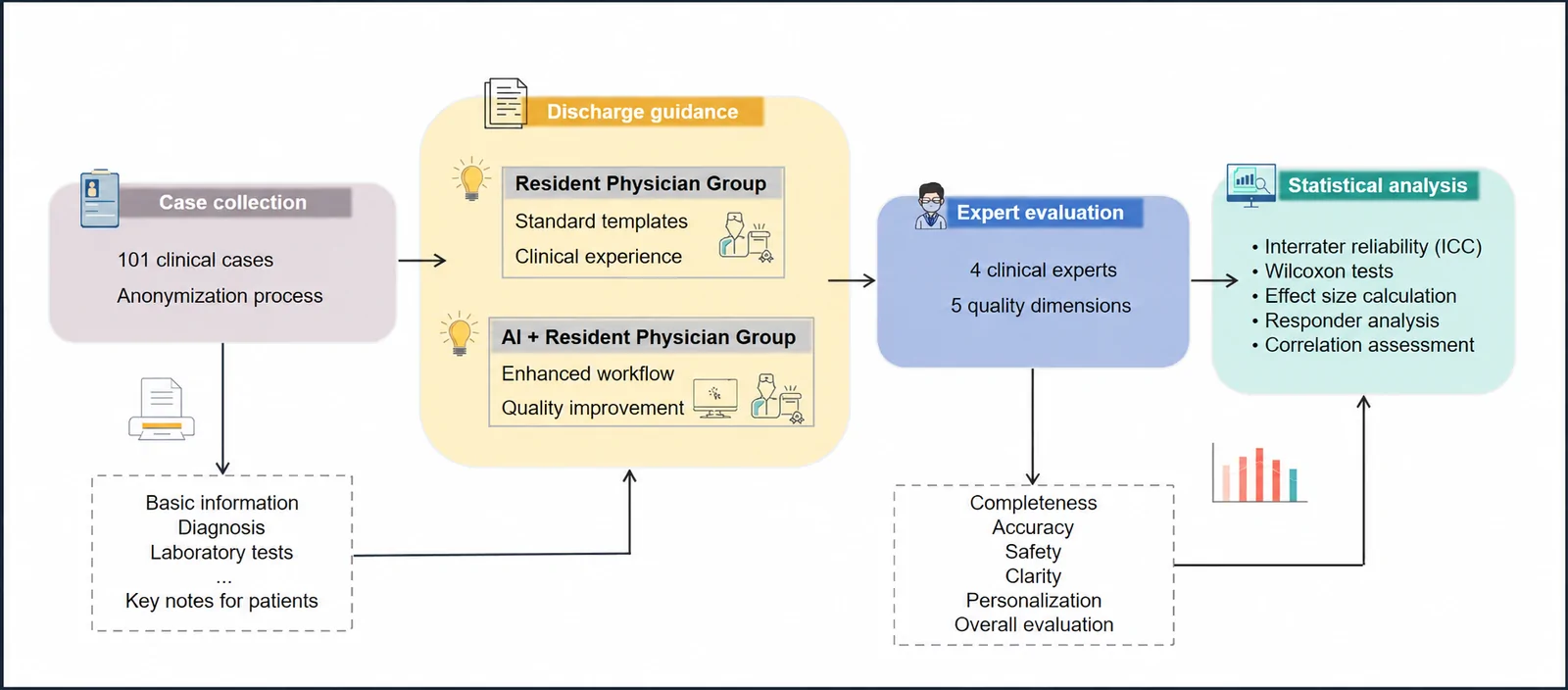
The workflow of this study. A total of 101 patients with diabetic kidney disease were retrospectively enrolled. Discharge instructions were generated independently by resident physicians and by another group of residents with the same level assisted by DeepSeek-V3.2. All documents were evaluated by 4 clinical experts across 5 dimensions using a 5-point Likert scale. Intergroup comparisons were performed using paired Wilcoxon signed-rank tests. ICC: intraclass correlation coefficient.

### Ethical Considerations

The research was approved by the Ethics Committee of Qilu Hospital of Shandong University (KYLL-2026-01-045). All procedures followed the ethical principles of the Declaration of Helsinki. Because all patient data were retrospectively retrieved and fully anonymized before analysis, the requirement for individual informed consent was waived by the board. The study strictly adhered to the Declaration of Helsinki and relevant JMIR editorial policies on research ethics and informed consent.

### Case Collection

This study collected 101 cases of patients with DKD from the hospital system between January 2023 and December 2025, encompassing patients at different stages of CKD. Collected information included patients’ basic personal details, disease diagnoses, complications, laboratory test results, and discharge instructions. The inclusion criteria were as follows: (1) primary discharge diagnosis of DKD, (2) discharge date between January 1, 2023, and December 31, 2025, (3) medical records containing sufficient key information to generate discharge instructions, and (4) clearly defined CKD stage. The study sample size was determined based on multiple statistical considerations. A total of 101 cases were included, with discharge instructions generated for each case by both the resident group and the AI-assisted group, yielding 202 discharge instructions. Four clinical experts evaluated each discharge instruction across 5 dimensions, yielding 808 assessment results (101 cases×2 groups×4 experts). Each result comprised 5 dimension scores, totaling 4040 data points (808×5). On the basis of pre-experimental data, the desired detection power was set with a standardized effect size of Cohen *d*=0.5 (moderate effect), a significance level of α=.05, statistical power of 0.8, and a minimum sample size of 34. Considering nonparametric test efficiency loss, reliable estimation of intrarater correlation coefficients for 4 evaluators, multiple comparisons correction across 5 dimensions (Bonferroni correction requiring a 25% increase in sample size), and a 10% data buffer, the minimum required sample size was calculated as 55 cases. A total of 101 cases were actually enrolled to ensure adequate statistical power and result reliability.

### Generation of Patient Discharge Instructions

First, we collected 101 patient discharge instructions written by various junior residents from the hospital system. The residents strictly adhered to actual clinical diagnoses and treatment plans when drafting these instructions. This study used the DeepSeek-V3.2 model via its API, using default generation parameters (temperature 1.0, maximum output length of 2000 tokens). After anonymizing the personal information of these 101 patients, the DeepSeek-V3.2 model generated 101 discharge instructions based on the patients’ clinical diagnoses and treatment plans. Residents reviewed 101 model-generated discharge instructions and finalized them.

The specific instructions were as follows:

As a professional and meticulous clinician, you are required to develop a structured, personalized, and actionable discharge instruction for the patient based on the provided ‘basic patient information, discharge diagnosis, comorbidities, test results during hospitalization, discharge orders, and additional patient care keywords.’ Please strictly adhere to the following principles: (1) The content must correspond to the patient’s specific condition, test results, risk factors, and medication regimen; do not invent conditions or treatment plans not mentioned during hospitalization. (2) If abnormal indicators or risk factors are present in the source data, include targeted recommendations in the discharge instructions. (3) If patient care keywords are provided, integrate these keywords into the discharge instructions as a personalized, supplementary section. (4) Discharge instructions must be clearly structured and expressed, with text complexity appropriate for a sixth-grade reading level; (5) Avoid making diagnostic speculations or providing uncertain medical information; (6) Do not use phrases such as “If you have ever...,” “As an AI,” or self-referential prompts. Please provide only professional content, but may offer compassionate advice as appropriate based on the patient’s condition.

### Expert Evaluation

This study invited chief physicians with at least 10 years of clinical experience as evaluators, including 2 nephrology specialists and 2 endocrinology specialists, to assess discharge instructions for the resident group and the AI-assisted group, respectively. The 5-dimension rating rubric was adapted from established discharge instruction and patient-education quality frameworks and finalized by expert consensus; its content validity was reviewed by all assessors during standardized training. Before formal evaluation, all assessors received standardized training covering Likert scale interpretation, dimension-specific scoring criteria, and preassessment exercises to ensure scoring consistency. A 5-point Likert scale was used to evaluate five dimensions as follows: (1) completeness—whether key points for discharge instructions for patients with DKD (eg, medication regimen, diet, fluid management, blood pressure and glucose control, follow-up appointments, and warning symptoms) were covered without significant omissions; (2) accuracy—consistency with patient medical records, CKD staging, laboratory tests, and treatment plans; absence of errors in dosage, frequency, or procedures; absence of recommendations inconsistent with clinical guidelines; (3) safety—presence of harmful advice or omitted potential risks in the text, such as incorrect indications for diuretic use, neglect of hyperkalemia risk, omission of heart failure volume management, disregard of hypoglycemia risk, or inappropriate dietary advice; (4) clarity—whether the language is logically coherent, well-organized, and easily understandable; and (5) personalization—whether personalized guidance is provided based on the patient’s disease stage, comorbidities (eg, hypertension, anemia, heart failure, and hyperkalemia risk), and test results.

### Statistical Analysis

Data analysis and graphing were performed using R software (version 4.5.2). The significance level was set at α=.05 (2-tailed). The Shapiro-Wilk test was applied to assess the normality of quantitative data. Normally distributed data were presented as mean (SD), whereas nonnormally distributed data were presented as median (IQR) and analyzed using nonparametric tests. Qualitative data were presented as frequency (percentage). The intraclass correlation coefficient (ICC) was used to evaluate consistency among the 4 expert scorers. ICC (2,1) was calculated based on a 2-way random-effects model. Interpretation criteria for ICC values were as follows: <0.40 indicated poor consistency, 0.40‐0.75 indicated moderate consistency, and ≥0.75 indicated excellent consistency. Due to nonnormal data distribution (confirmed by the Shapiro-Wilk test), paired Wilcoxon signed-rank tests were used for intergroup comparisons. To control for multiple comparisons, the Bonferroni correction was applied (adjusted α=.05/6=.0083). Effect sizes were calculated using the formula *r*=Z/√N and interpreted according to Cohen criteria: 0.1 (small effect), 0.3 (moderate effect), and 0.5 (large effect). Intergroup comparisons were conducted for different CKD stages and dimensions (Wilcoxon signed-rank test), with Bonferroni correction applied for multiple comparisons. Spearman rank correlation coefficients between CKD stages (treated as ordered variables 1‐5) and assessment dimension scores across groups were calculated. Correlation strength was classified as follows: |ρ|<0.2 (very weak), 0.2‐0.4 (weak), 0.4‐0.6 (moderate), 0.6‐0.8 (strong), and ≥0.8 (extremely strong). Cases with clinically meaningful improvement were identified as those showing a total score increase of ≥1 point in the AI-assisted group compared with the resident group. The proportion of responders and its 95% CI were calculated using the Clopper-Pearson exact method. A priori, an increase of ≥1 point on the 5-point Likert scale was defined as a clinically meaningful improvement. This threshold was selected as a conservative and clinically interpretable criterion, corresponding to a full 1-category shift in the overall quality rating. Because there is no universally applicable minimal important difference for all ordinal rating scales, the ≥1-point threshold was defined specifically for this study to represent a readily interpretable and substantial change in overall document quality, rather than as a universally validated minimal clinically important difference [16-18].

## Results

### Case Information

This study retrospectively collected 101 cases of patients with DKD from a tertiary hospital system to generate 202 discharge instructions for 2 groups (resident group and AI-assisted group). Case characteristics are shown in Table 1. The mean age of patients in these cases was 57.9 (SD 13.6) years. Among the 101 patients, 66 (65.3%) were male, and 35 (34.7%) were female. Twenty-two patients were obese. Cases included patients with CKD stages 1 to 5. There were 6 (5.9%) patients with CKD stage 1, 10 (9.9%) with CKD stage 2, 18 (17.8%) with CKD stage 3, 20 (19.8%) with CKD stage 4, and 47 (46.5%) with CKD stage 5. One patient had no complications, 23 patients had 1 to 3 complications, and 77 patients had 3 or more complications.

**Table 1.** Basic case information (n=101)^a^.

	Female patients, n (%)	Male patients, n (%)	Total, n (%)
BMI (kg/m^2^)			
≥28	10(9.9)	12 (11.9)	22 (21.8)
<28	25 (24.8)	54 (53.5)	79 (78.2)
Proteinuria			
Microalbuminuria (150‐300 mg/24 h)	1 (1.0)	1 (1.0)	2 (2.0)
Mild proteinuria (300 mg-1 g/24 h)	4 (4.0)	3 (3.0)	7 (6.9)
Moderate proteinuria (1‐3.5 g/24 h)	6 (5.9)	24 (23.8)	30 (29.7)
Massive proteinuria (>3.5 g/24 h)	11(10.9)	15 (14.9)	26 (25.7)
Hypoalbuminemia	10(9.9)	8 (7.9)	18 (17.8)
Chronic kidney disease stage			
Stage 1	1 (1.0)	5 (5.0)	6 (5.9)
Stage 2	2 (2.0)	8 (7.9)	10 (9.9)
Stage 3	7 (6.9)	11 (10.9)	18 (17.8)
Stage 4	8 (7.0)	12 (11.9)	20 (19.8)
Stage 5	17(16.8)	30 (29.7)	47 (46.5)
Diabetes complications			
Diabetic peripheral neuropathy	5 (5.0)	14 (13.9)	19 (18.8)
Diabetic retinopathy	11 (10.9)	19 (18.8)	30 (29.7)
Diabetic foot	0 (0.0)	3 (3.0)	3 (3.0)
Diabetic peripheral vascular disease	1 (1.0)	6 (5.9)	7 (6.9)
Comorbidities			
Hypertension	29 (28.7)	55 (54.5)	84 (83.2)
Hyperlipidemia	7 (6.9)	8 (7.9)	15 (14.9)
Hyperuricemia	24 (23.8)	46 (45.5)	70 (69.3)
Hypocalcemia	2 (2.0)	3 (3.0)	5 (5.0)
Hyperphosphatemia	1 (1.0)	28 (27.7)	29 (28.7)
Renal osteopathy	2 (2.0)	5 (5.0)	7 (6.9)
Dyskalemia			
Hyperkalemia	1 (1.0)	1(1.0)	2 (2.0)
Hypokalemia	1 (1.0)	2 (2.0)	3 (3.0)
Anemia	29 (28.7)	50 (49.5)	79 (78.2)
Hypothyroidism	6 (5.9)	6 (5.9)	12 (11.9)
Coronary atherosclerotic heart disease	12 (11.9)	8 (7.9)	20 (19.8)
Heart failure	16 (15.8)	29 (28.7)	45 (44.6)
Dialysis	6 (5.9)	6 (5.9)	12 (11.9)

a The mean age of the patients was 57.9 (SD 13.6) years. Of the 101 patients, 66 (65.3%) were male and 35 (34.7%) were female.

### Expert Scoring Consistency

The consistency analysis of the 4 expert ratings is shown in Table 2. The ICC for the resident group was 0.817 (95% CI 0.763‐0.864; *F*_100,300_=18.876; *P*<.001), with an agreement ICC of 0.776. The ICC for the AI-assisted group was 0.860 (95% CI 0.816‐0.897; *F*_100,300_=25.531; *P*<.001), with an agreement ICC of 0.842. Both groups demonstrated excellent interrater consistency.

**Table 2.** Intraclass correlation coefficient (ICC) analysis results.

Group	ICC (consistency; 95% CI)	ICC (agreement)	*F* test (*df*)	*P* value
Resident group	0.817 (0.763-0.864)	0.776	18.876 (100, 300)	<.001
AI-assisted group	0.860 (0.816-0.897)	0.842	25.531 (100, 300)	<.001

### Comparison of Discharge Instructions Between Resident Group and AI-Assisted Group Across Dimensions

The expert evaluation results are shown in Figure 2. The Bonferroni-corrected Wilcoxon signed-rank test revealed that the median scores of the AI-assisted group were substantially higher than those of the traditional resident group across all 6 dimensions: completeness, accuracy, safety, clarity, personalization, and overall evaluation (all *P*<.001). Notably, the test statistic *W* was 0 for all dimensions, indicating that across all paired samples, the AI-assisted group’s scores were not lower than those of the traditional resident group and were higher in the vast majority of cases. Combined with large effect sizes (*r* values ranging from 0.741-0.927), these results demonstrated that using AI-assisted tools to generate discharge instructions in this study significantly improved discharge instruction quality. As shown in Figure 2, experts perceived the resident group’s performance as relatively stronger in accuracy and safety compared with other dimensions, with no significant issues in these areas. The AI-assisted group demonstrated consistent performance across dimensions, maintaining generally high expert ratings. As shown in Figure 3, the distribution of differences across all dimensions was entirely skewed toward positive values, with no instances of lower scores for the AI group. This aligned with the *W*=0 result from the Wilcoxon signed-rank test.

**Figure 2.**
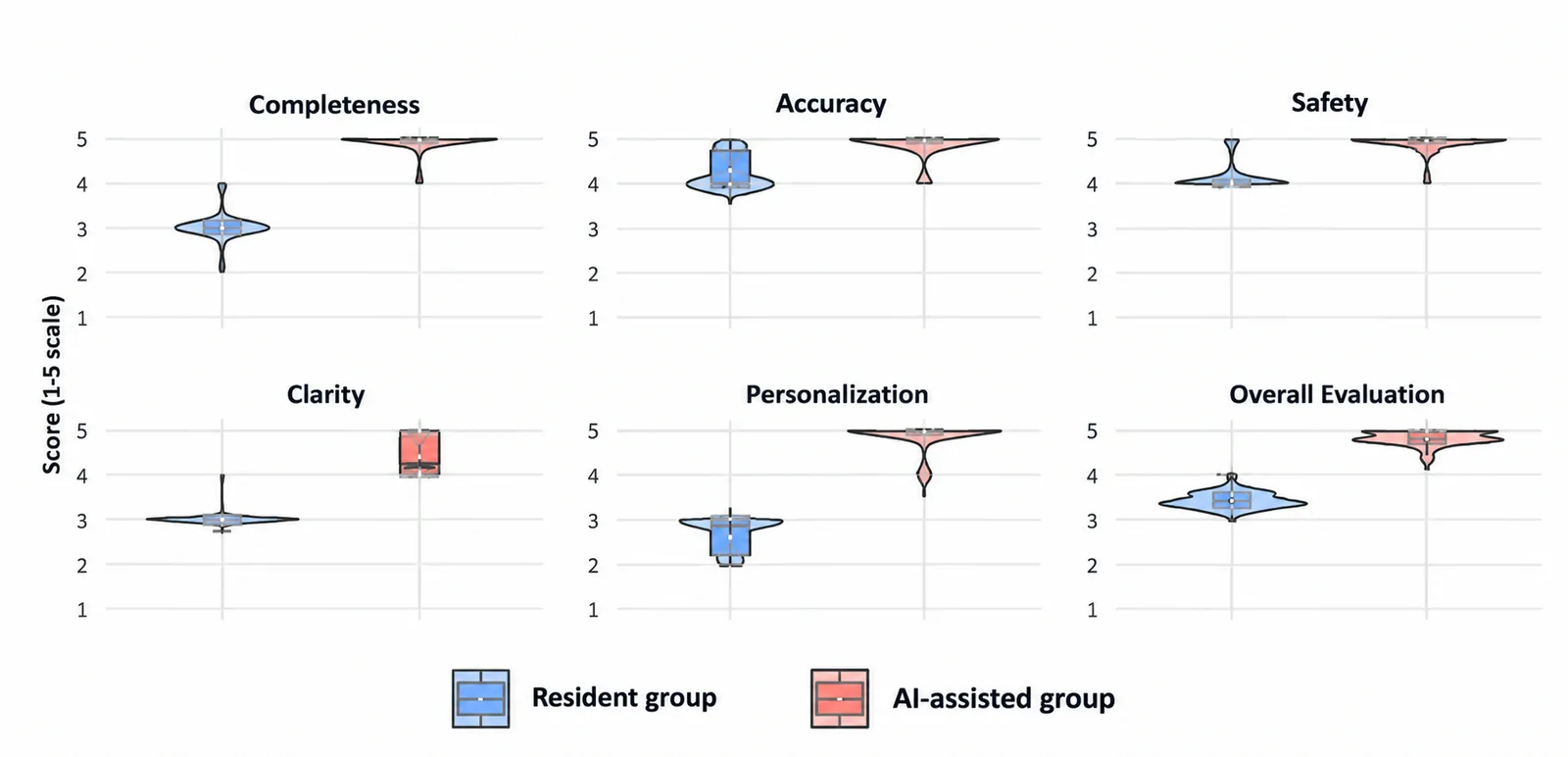
Comparison of expert scores between the resident group and the AI-assisted group across different evaluation dimensions. The evaluation criteria consist of 5 dimensions: completeness, accuracy, safety, clarity, and personalization. In addition, there is an overall evaluation (average score). Box plots show medians and IQRs. All comparisons were statistically significant (Bonferroni-adjusted *P*<.001), with effect sizes (*r*) ranging from 0.741 to 0.927.

**Figure 3.**
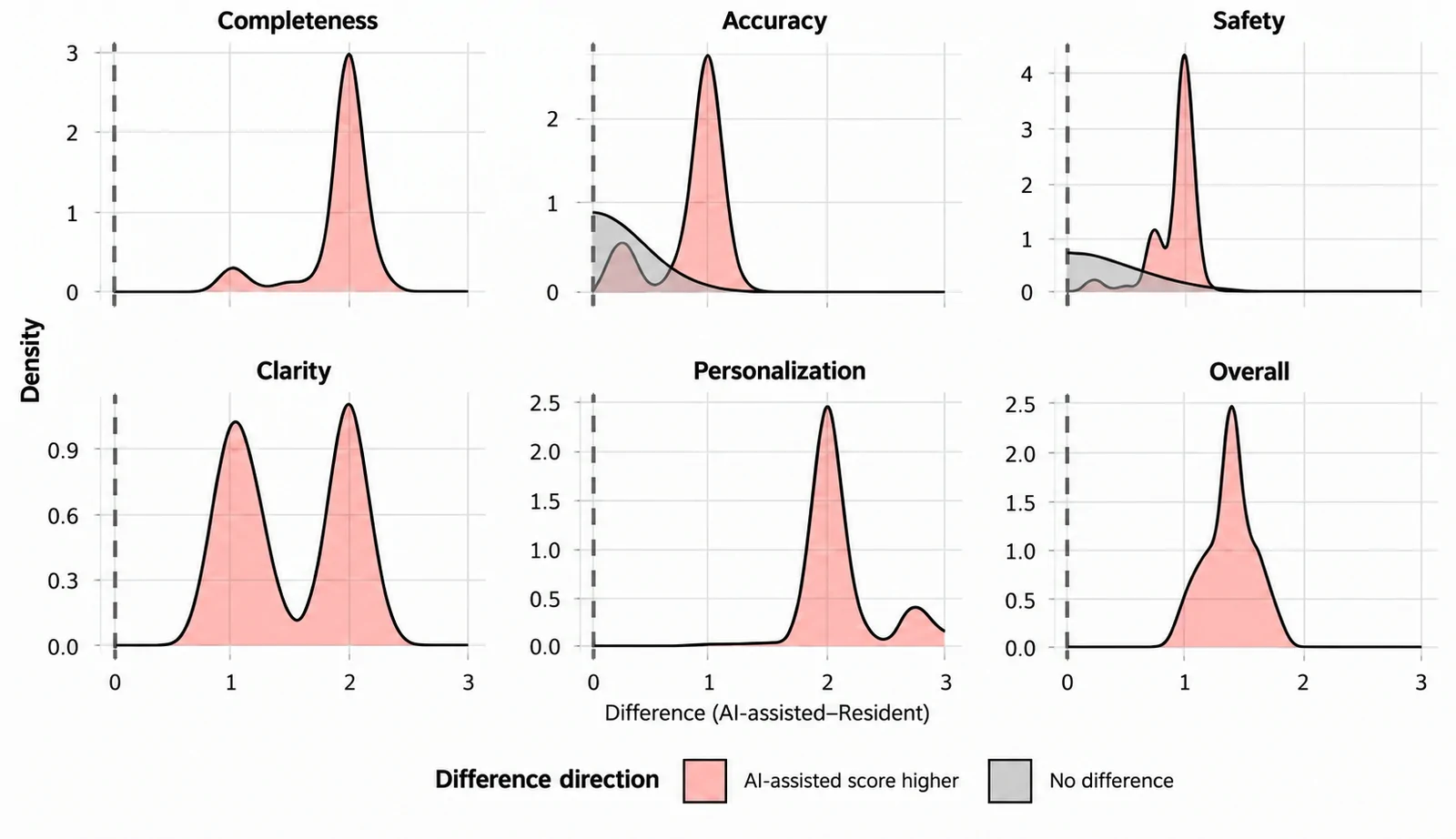
Distribution of score differences between groups. Score differences: AI-assisted group minus resident group. Positive values indicate higher scores in the AI-assisted group. Density curves illustrate the frequency of difference magnitudes. No paired sample showed a lower score in the AI-assisted group, consistent with Wilcoxon signed-rank test results (*W*=0 for all dimensions).

### Comparison of Discharge Instructions Between Resident Group and AI-Assisted Group Across Different CKD Stages

This study conducted a correlation analysis between CKD stage and performance across different assessment dimensions, with results shown in Figure 4. In the traditional resident group, CKD stage showed weak negative correlations with multiple assessment dimensions: Spearman correlation coefficients (ρ) for the 4 core dimensions—accuracy, safety, clarity, and overall evaluation—ranged from –0.33 to –0.37 (*P*<.001), all exhibiting significant statistical differences. As the patients’ CKD stage increased (indicating more severe and complex conditions), scores for accuracy, clarity, safety, and overall quality of discharge instructions completed independently by residents showed a declining trend. For other dimensions, such as completeness and personalization, only very weak and statistically insignificant correlations existed with CKD stage (|ρ|<0.2; *P*>.05). In the AI-assisted group, CKD stage showed no significant association with most evaluation dimensions: a weak positive correlation existed for accuracy (ρ=0.247; *P*=.01), while the absolute values of correlation coefficients ρ for the remaining 5 dimensions (completeness, safety, clarity, personalization, and overall evaluation) were all less than 0.15 (|ρ|<0.15). All corresponding *P* values exceeded .05, indicating no statistical significance. With AI assistance, quality scores across all dimensions of discharge instructions no longer showed significant variation with increasing CKD stage, maintaining consistent quality across cases of varying complexity.

**Figure 4.**
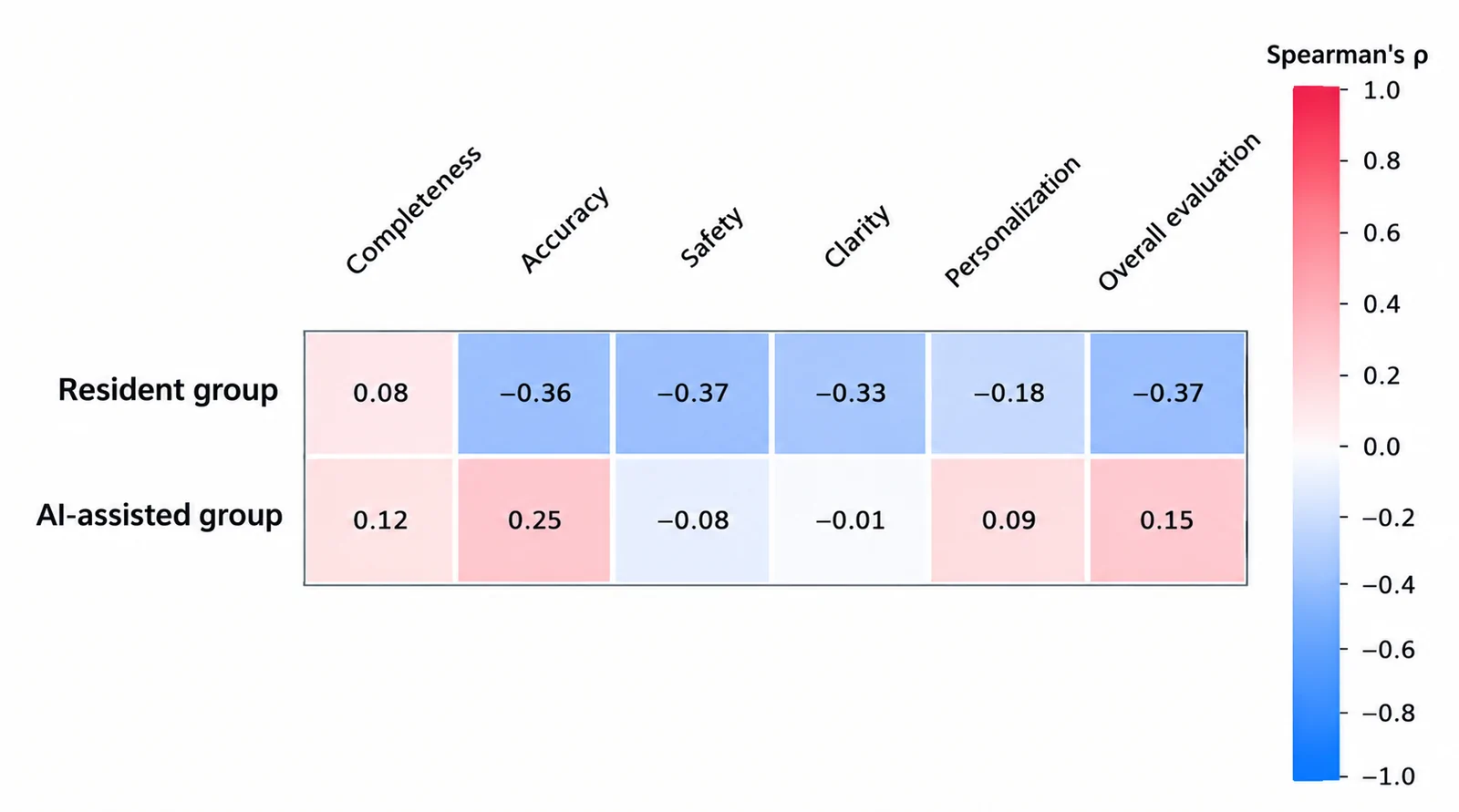
Heatmap of Spearman correlation coefficients between chronic kidney disease (CKD) stage and evaluation dimensions in both groups. In the resident group, CKD stage showed weak negative correlations with accuracy, safety, clarity, and overall scores (ρ=−0.33 to −0.37; *P*<.001). In the AI-assisted group, no significant correlations were observed (|ρ|<0.15; *P*>.05 for most dimensions). Correlation strength: |ρ|<0.2 (very weak), 0.2‐0.4 (weak), 0.4‐0.6 (moderate), 0.6‐0.8 (strong), ≥0.8 (very strong).

Subsequently, this study compared the resident group with the AI-assisted group across different CKD stages and assessment dimensions, with results shown in Figure 5. In the CKD stage 1 (early) case subgroup, the AI-assisted group exhibited higher median scores than the resident group across most dimensions (completeness, clarity, personalization, and overall evaluation) (*P*<.05). However, after correction for multiple comparisons, none of these differences reached statistical significance (adjusted *P*>.05). In simple cases, the advantage of AI assistance remained inconsistent. In CKD stage 2, as disease progression increased, the advantage of AI assistance became apparent. The AI-assisted group scored significantly higher than the resident group across the following evaluations—completeness, safety, clarity, personalization, and overall evaluation (adjusted *P*<.01), with effect sizes ranging from *r*=0.840 to 0.910. Both groups performed comparably in content accuracy. In more complex cases involving CKD stages 3 to 5 (mid-to-late stages), AI-assisted evaluation demonstrated comprehensive and consistent advantages. For CKD stage 3, significant differences existed across all dimensions except accuracy, as well as in overall evaluation (adjusted *P*<.001), with effect sizes ranging from *r*=0.864 to 0.958. For CKD stages 4 and 5, scores for the AI-assisted group were significantly higher than those of the resident group across all 5 dimensions and overall evaluation (all adjusted *P*<.001), with effect sizes ranging from 0.705 to 0.947. The enhancement efficacy of AI assistance positively correlated with case complexity. Particularly in complex mid-to-late stage cases, AI assistance significantly improved the quality of discharge instructions.

**Figure 5.**
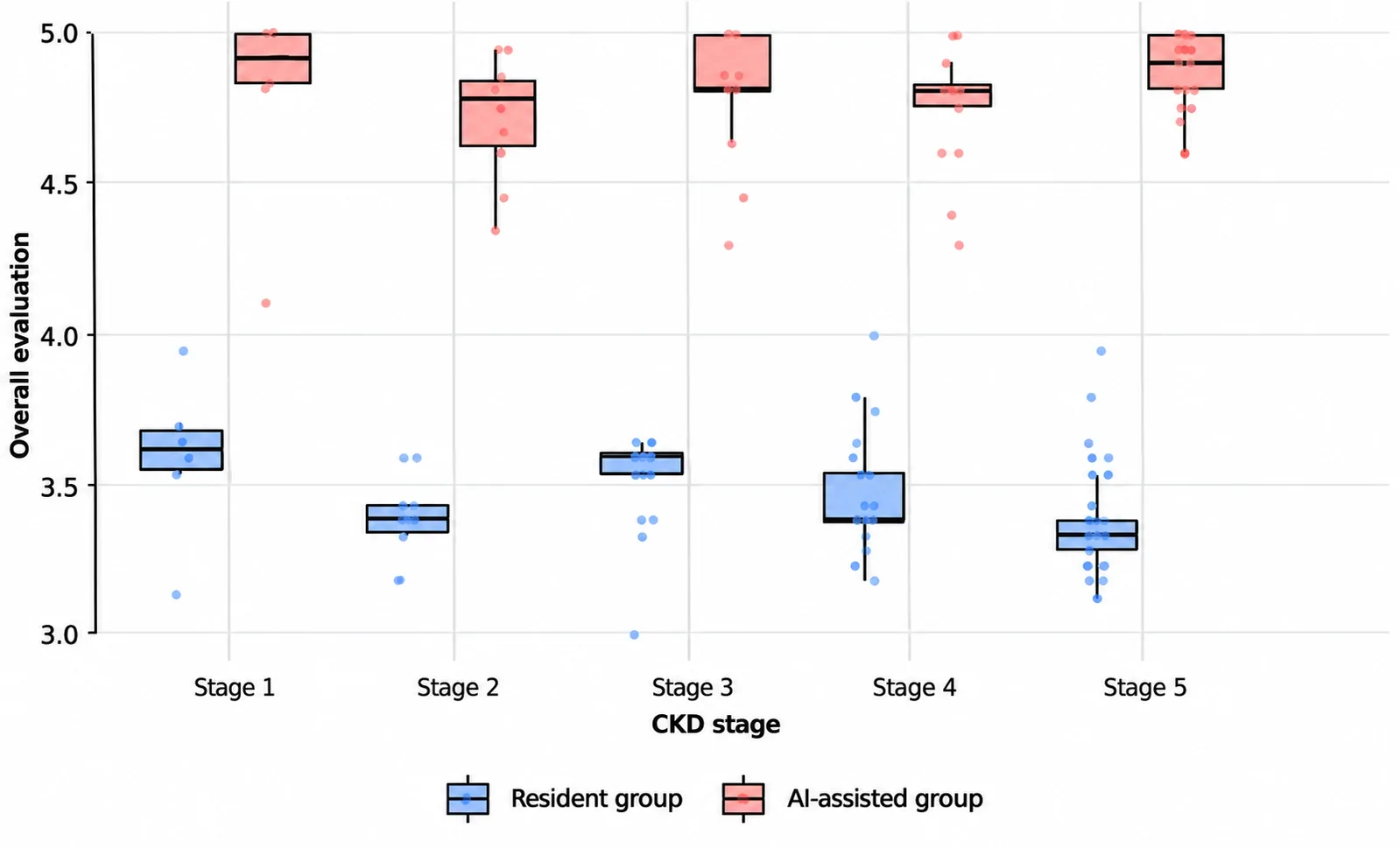
Distribution of scores across 2 groups at different chronic kidney disease (CKD) stages. Box plots represent medians and IQRs.

### Expert-Rated Responder Analysis Results

Using the prespecified threshold of a ≥1-point increase in the overall score, 100 (99%) of 101 cases were classified as responders following AI assistance (95% CI 94.6%‐100.0%), while 1 case (1%) was classified as a nonresponder. Thus, nearly all cases demonstrated a 1-category or greater improvement in the overall expert-rated quality score after AI assistance.

## Discussion

### Principal Findings

This study evaluated whether LLM assistance could improve the quality of DKD discharge instructions produced by junior physicians. Across 101 patient cases and 202 discharge instructions, the AI-assisted group achieved significantly higher expert-rated scores than the resident group across all 6 evaluated dimensions: completeness, accuracy, safety, clarity, personalization, and overall quality (all Bonferroni-adjusted *P*<.001), with large effect sizes (*r*=0.741‐0.927). Interrater reliability was excellent in both groups, with ICCs of 0.817 (95% CI 0.763‐0.864) and 0.860 (95% CI 0.816‐0.897), respectively. The benefit of AI assistance was particularly evident as CKD stage increased. In the resident group, advancing CKD stage was associated with lower scores for accuracy, safety, clarity, and overall quality, whereas these associations were largely absent in the AI-assisted group. The responder analysis further showed that 99% (100/101; 95% CI 94.6%‐100.0%) of cases achieved the prespecified ≥1-point improvement in overall quality. Taken together, these findings suggest that LLM assistance may help junior physicians produce more comprehensive and consistently structured discharge instructions, particularly for clinically complex DKD cases.

### Interpretation and Comparison With Previous Studies

The observed improvement across multiple dimensions is consistent with previous research indicating that generative AI can improve the organization, readability, and patient-centered presentation of medical information [12-15]. Zaretsky et al [13] demonstrated that generative AI could transform inpatient discharge summaries into more patient-friendly language and formats, while Stanceski et al [14] reported that generative AI could produce patient-centered discharge instructions with generally favorable quality and safety characteristics. Williams et al [15] further showed that physician- and LLM-generated hospital discharge summaries could achieve comparable performance across several evaluated characteristics. Our study extends this literature by examining a human-AI collaborative workflow rather than comparing physicians with fully autonomous AI-generated documents. In the present workflow, the LLM generated an initial draft based on patient-specific clinical information, after which junior physicians reviewed and finalized the content. Thus, the observed differences reflect the performance of an assistive documentation process rather than that of an autonomous LLM.

The greater benefit observed in advanced CKD is clinically relevant. Patients with DKD with more advanced CKD frequently have multiple concurrent management requirements, including anemia, electrolyte abnormalities, cardiovascular disease, volume management, dietary restrictions, and preparation for or receipt of kidney replacement therapy. As the number and interdependence of these requirements increase, discharge instructions become more difficult to organize and personalize. In our study, resident-only instructions showed a progressive decline in accuracy, safety, clarity, and overall quality with increasing CKD stage. In contrast, most dimensions of the AI-assisted instructions were not significantly associated with CKD stage. This pattern suggests that LLM assistance may partially mitigate the effect of increasing information complexity on the consistency of discharge documentation. However, this interpretation should be considered hypothesis-generating because the present study did not directly measure the mechanisms through which AI assistance affected physician performance.

One possible explanation is that LLM assistance may reduce the cognitive effort required for information organization and language formulation. Cognitive load theory proposes that complex tasks can impose substantial demands on working memory when multiple sources of information must be processed simultaneously [19,20]. In the present workflow, the LLM was used primarily to organize patient-specific information and generate a structured draft, while residents retained responsibility for clinical review and finalization. This division of labor may allow clinicians to devote greater attention to checking patient-specific risks, medication instructions, and follow-up recommendations rather than constructing the entire document from the beginning. This interpretation is also consistent with recent systematic evidence suggesting that LLMs may support clinical documentation and communication workflows, while emphasizing the importance of human oversight, standardized evaluation, and external validation [9,10]. Importantly, our findings should not be interpreted as evidence that LLMs can independently generate safe discharge instructions. Rather, they support further evaluation of a workflow in which AI assists with drafting while clinicians remain responsible for verification and final clinical decisions.

The responder analysis showed that 100 of 101 cases met the prespecified criterion of a ≥1-point improvement in overall expert-rated quality. This high proportion indicates that the observed improvement was not restricted to a small subset of cases. However, the threshold used in this study was specifically defined for this ordinal rating framework and should not be interpreted as a universally validated minimal clinically important difference. Moreover, because the outcome was expert-rated document quality rather than a patient-centered clinical outcome, the responder analysis should be regarded as an indicator of document-level improvement rather than evidence of improved patient health outcomes.

### Limitations

Several limitations should be considered. First, the study was retrospective and conducted at a single tertiary hospital, which may limit the generalizability of the findings to other institutions, specialties, health care systems, and physician experience levels. Second, the 4 expert evaluators were not blinded to group assignment. Because the AI-assisted documents had a recognizable structured and patient-oriented format, evaluators may have been able to identify the intervention condition, introducing the possibility of assessment bias. Third, although the same 101 patient cases were used for both groups, the documents were generated by 2 separate groups of junior residents. Differences in individual writing ability, clinical experience, or documentation style between resident groups could therefore have contributed to the observed differences. Fourth, the study evaluated document quality rather than downstream patient-centered outcomes. Patient comprehension, medication adherence, self-management behavior, follow-up attendance, readmission, and other clinical outcomes were not assessed. Fifth, documentation time, physician workload, user satisfaction, and the amount of physician editing required after AI generation were not systematically recorded. Therefore, the potential of this workflow to reduce documentation burden remains to be demonstrated rather than established. Finally, although expert-rated safety was included as one of the evaluation dimensions, the study was not designed to establish clinical safety or detect adverse events associated with AI-assisted discharge instructions.

### Conclusions and Broader Implications

In this single-center retrospective study, LLM assistance was associated with significantly higher expert-rated quality of DKD discharge instructions produced by junior physicians, with the largest relative advantages observed in patients with more advanced CKD. The findings suggest that a human-AI collaborative workflow, in which an LLM generates a patient-specific draft and a clinician reviews and finalizes the content, may provide a feasible approach to improving the consistency and organization of discharge documentation. However, these findings should not be interpreted as evidence that LLMs can replace clinical judgment or independently ensure the safety of discharge instructions. Future prospective, multicenter studies should evaluate the reproducibility of these findings across institutions and physician groups and should incorporate objective measures of documentation time, physician workload, patient comprehension, adherence, and postdischarge clinical outcomes. Such studies will be important for determining whether improvements in document quality translate into meaningful benefits during the transition from hospital-based to long-term DKD care.

## Abbreviations

CKD: chronic kidney disease
DKD: diabetic kidney disease
ICC: intraclass correlation coefficient
LLM: large language model

## References

[1] Hoogeveen EK (2022). The epidemiology of diabetic kidney disease. Kidney Dial.

[2] GBD Chronic Kidney Disease Collaboration (2020). Global, regional, and national burden of chronic kidney disease, 1990-2017: a systematic analysis for the Global Burden of Disease Study 2017. Lancet.

[3] Afkarian M, Sachs MC, Kestenbaum B (2013). Kidney disease and increased mortality risk in type 2 diabetes. J Am Soc Nephrol.

[4] Kidney Disease: Improving Global Outcomes (KDIGO) CKD Work Group (2024). KDIGO 2024 clinical practice guideline for the evaluation and management of chronic kidney disease. Kidney Int.

[5] American Diabetes Association Professional Practice Committee (2025). 11. Chronic kidney disease and risk management: Standards of Care in Diabetes-2025. Diabetes Care.

[6] Kripalani S, LeFevre F, Phillips CO, Williams MV, Basaviah P, Baker DW (2007). Deficits in communication and information transfer between hospital-based and primary care physicians: implications for patient safety and continuity of care. JAMA.

[7] Jack BW, Chetty VK, Anthony D (2009). A reengineered hospital discharge program to decrease rehospitalization: a randomized trial. Ann Intern Med.

[8] Hesselink G, Zegers M, Vernooij-Dassen M (2014). Improving patient discharge and reducing hospital readmissions by using Intervention Mapping. BMC Health Serv Res.

[9] Artsi Y, Sorin V, Glicksberg BS, Korfiatis P, Nadkarni GN, Klang E (2025). Large language models in real-world clinical workflows: a systematic review of applications and implementation. Front Digit Health.

[10] Sabanayagam C, Banu R, Lim C (2025). Artificial intelligence in chronic kidney disease management: a scoping review. Theranostics.

[11] Arndt BG, Beasley JW, Watkinson MD (2017). Tethered to the EHR: primary care physician workload assessment using EHR event log data and time-motion observations. Ann Fam Med.

[12] Ayers JW, Poliak A, Dredze M (2023). Comparing physician and artificial intelligence chatbot responses to patient questions posted to a public social media forum. JAMA Intern Med.

[13] Zaretsky J, Kim JM, Baskharoun S (2024). Generative artificial intelligence to transform inpatient discharge summaries to patient-friendly language and format. JAMA Netw Open.

[14] Stanceski K, Zhong S, Zhang X (2024). The quality and safety of using generative AI to produce patient-centred discharge instructions. NPJ Digit Med.

[15] Williams CY, Subramanian CR, Ali SS (2025). Physician- and large language model-generated hospital discharge summaries. JAMA Intern Med.

[16] Jaeschke R, Singer J, Guyatt GH (1989). Measurement of health status. Ascertaining the minimal clinically important difference. Control Clin Trials.

[17] de Vet HC, Terwee CB, Ostelo RW, Beckerman H, Knol DL, Bouter LM (2006). Minimal changes in health status questionnaires: distinction between minimally detectable change and minimally important change. Health Qual Life Outcomes.

[18] Terwee CB, Peipert JD, Chapman R (2021). Minimal important change (MIC): a conceptual clarification and systematic review of MIC estimates of PROMIS measures. Qual Life Res.

[19] Sweller J (1988). Cognitive load during problem solving: effects on learning. Cogn Sci.

[20] Sweller J (2011). Psychology of Learning and Motivation.

